# Supplementary figures and images for: Oropharyngeal microbiome dysbiosis in esophageal squamous cell carcinoma: taxonomic shifts, metabolic reprogramming, and geographic disparities in a high-incidence cohort
Source: PeerJ. 2025 Oct 6;13:e20009. doi: 10.7717/peerj.20009 (PMC12510252; doi:10.7717/peerj.20009)

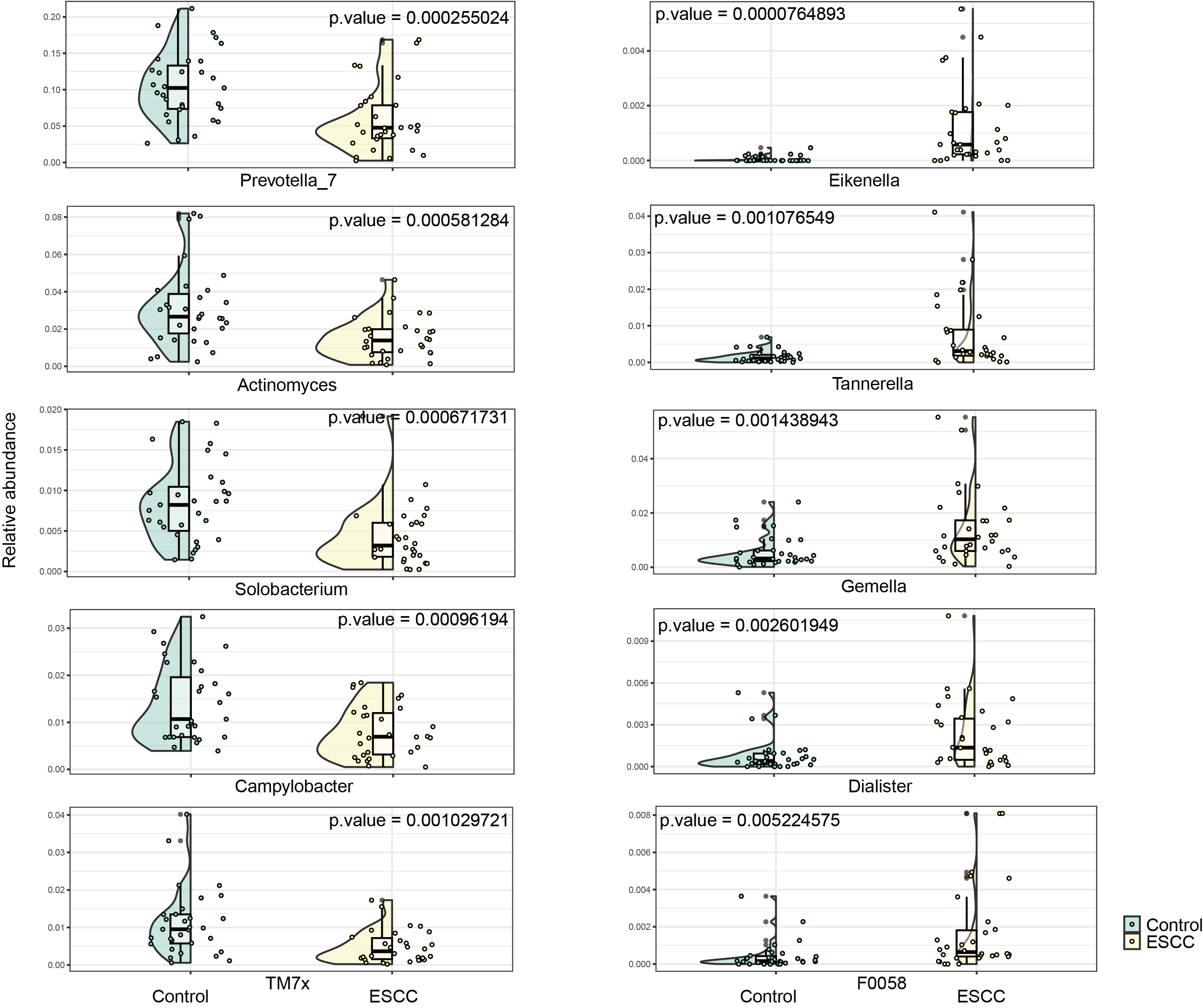

Supplement: Supplemental Information 1 — The left panel displays microbial genera exhibiting significantly higher abundance in the control group relative to the ESCC group, whereas the right panel presents genera that are significantly more abundant in the cancer group compared to healthy controls. Both panels are arranged in descending order based on statistical significance (p-value). [file peerj-13-20009-s001.png]
